# Supplementary material for: Characterization of Prenylated C-terminal Peptides Using a Thiopropyl-based Capture Technique and LC-MS/MS
Source: Mol Cell Proteomics. 2020 Apr 13;19(6):1005–16. doi: 10.1074/mcp.RA120.001944 (PMC7261820; doi:10.1074/mcp.RA120.001944)
Supplement: Figure S1: Extraction of prenyl proteins from mouse brain [file 158251_1_supp_495479_q7f3jm.docx]

**Figure S1. Extraction of prenyl proteins from mouse brain**

**Mouse Brains

Homogenize in 0.01N HCl

Centrifuge Suspension at 20,000 x g 10 min, 4 degrees C

Resuspend Pellet, Wash pellet 2x with 0.01N HCl as above

Resuspend Pellet in Extraction Buffer; incubate 1.5hr, 4 degrees C

Centrifuge Suspension at 20,000 x g 10 min, 4 degrees C

Save Supernatant; resuspend pellet in extraction buffer

Repeat extraction of pellet as above for 1.5 hr

Centrifuge Suspension at 20,000 x g 10 min, 4 degrees C

Combine Supernatant with 1st extract ; discard pellet

Add 10 mM TCEP to combined supernatant; incubate with mixing 1hr, 4 degrees C

Add 10mM iodoacetamide; incubate 30 min, room temperature

Centrifuge Suspension at 20,000 x g 10 min, 4 degrees C

Discard supernatant, resuspend pellet in 5 ml 8M Urea, 50mM ammonium bicarbonate

Remove residual TCEP/iodoacetamide by gel permeation chromatography on Sephadex G-25**
