# Supplementary material for: Characterization of Prenylated C-terminal Peptides Using a Thiopropyl-based Capture Technique and LC-MS/MS
Source: Mol Cell Proteomics. 2020 Apr 13;19(6):1005–16. doi: 10.1074/mcp.RA120.001944 (PMC7261820; doi:10.1074/mcp.RA120.001944)
Supplement: Figure S2: Prenyl protein and peptide capture and elution [file 158251_1_supp_495480_q7f3jn.docx]

**Figure S2. Prenyl protein and peptide capture and elution**

**Suspend thirty-five mg of lyophilized thiopropyl Sepharose resin in 1ml of water**

**Gently mix for 10 minutes.**

**Wash resin in a spin column using 5 rinses of 0.5 ml water followed by 5 rinses of 0.5ml of 50mM ABC. For each wash, thoroughly resuspend resin before centrifugation**

**Mix resin with 1mg of the urea-containing protein solution from the G25 column**

**Incubate suspension with end-over-end mixing for 18 hr at 4 degrees C.**

**Remove unbound proteins from the thiopropyl resin in spin column**

**Wash resin in the spin column using 0.5ml volumes (five washes with each) of the following: 6M urea/50mM ABC; 2M NaCl; 70% acetonitrile/ 0.1% formic acid in water; water, 50mM ABC.**

**Resuspend washed resin in 200 microliters of 50mM ABC.**

**Add one microgram of chymotrypsin (Promega sequencing grade) to the suspension**

**Incubate the mixture with vigorous mixing to maintain the resin in suspension at 37°C for 5-6 hours.**

**Place resin in the spin column and isolate unbound chymotryptic peptides**

**Wash resin with 100 microliters of 0.1% formic acid**

**Combine the wash was with the released peptides. Save unbound chymotryptic peptides for analysis.**

**DTT elution: resuspend resin in 200 microliters 50mM ABC containing 10mM DTT; incubate 2 hours at room temperature with vigorous mixing. Separate resin on spin column.**

**Rinse resin with 100 microliters of 0.1% formic acid in water (combine with the DTT- eluted peptides).**

**50% ACN elution:Suspend resin in 200 microliters of 50% acetonitrile/50% water containing 0.1% formic acid and incubate vigorously as above; mix overnight at room temperature.**

**Rinse resin with 100ul of the 50% acetonitrile solution and combine rinse with the eluted peptides from this step.**

**Dry peptides from the DTT and 50% ACN elutions under vacuum and resuspended dry material from each fraction in 50 microliters of 1 % formic acid in water. Purify peptides by removing salt and reagents using C18 containing pipet tips (OMIX, Agilent) and elute from the tips using 50% acetonitrile, 0.1% formic acid in water. Dry eluted peptides under vacuum and resuspend in 10% acetonitrile, 0.1% formic acid in water for analysis by LCMS.**
