# Supplementary material for: Characterization of Prenylated C-terminal Peptides Using a Thiopropyl-based Capture Technique and LC-MS/MS
Source: Mol Cell Proteomics. 2020 Apr 13;19(6):1005–16. doi: 10.1074/mcp.RA120.001944 (PMC7261820; doi:10.1074/mcp.RA120.001944)
Supplement: Figure S3: msms spectrum of c-terminal peptide from Rho-G [file 158251_1_supp_495481_q7f3jn.pdf]

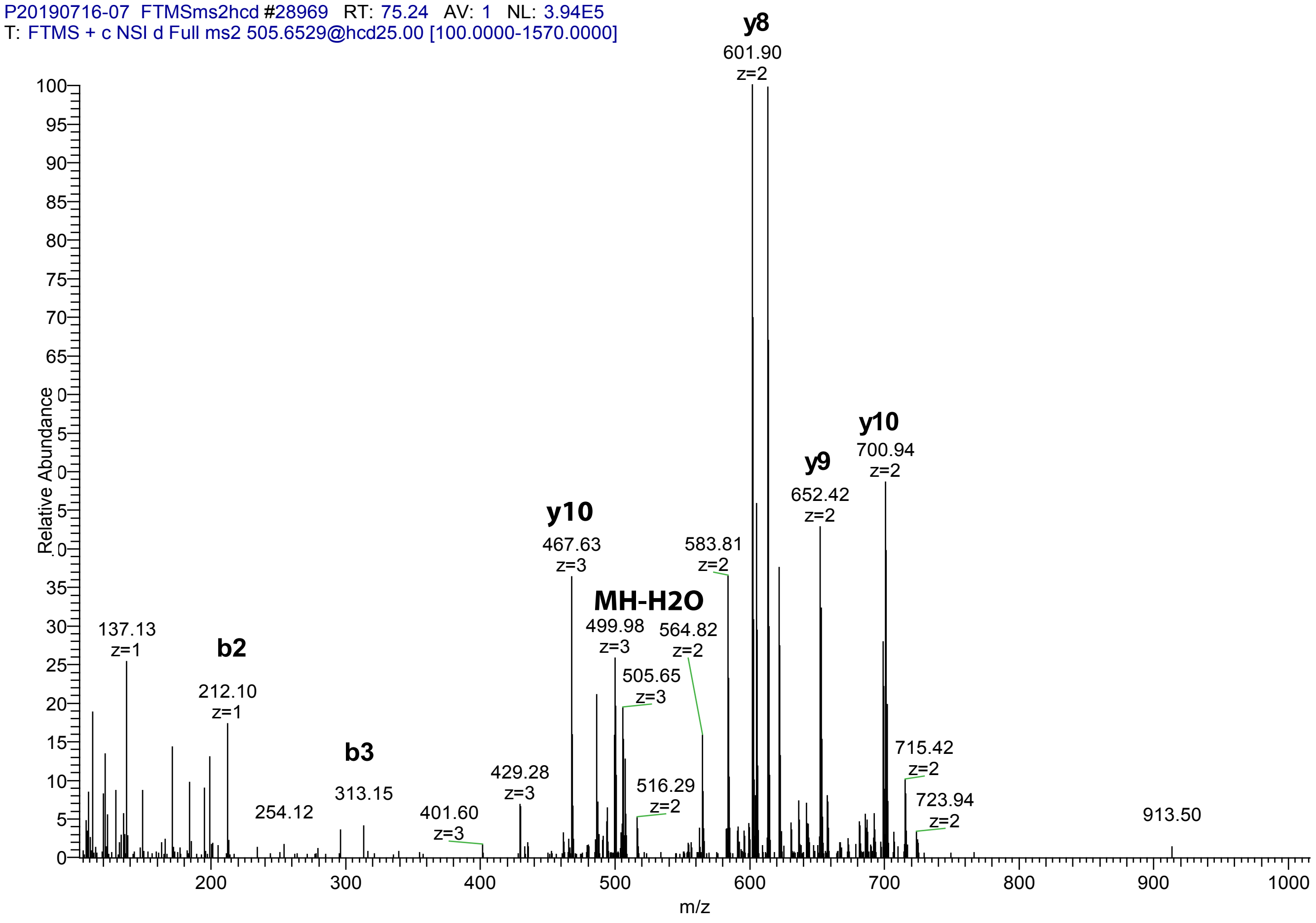

Figure S3. MS2 of **Rho G** c-terminal peptide (L)NPTPIKRGRSC(GeranylGeranyl)-Methyl+3, M+3H+=505.6529 from ms filter. Fragment masses are as follows (fragment:charge:mass): b2:+1: 212.10, b3:+1: 313.15, y8:+2: 601.89, y9:+2: 652.42, y10:+2: 700.94, y10:+3: 467.63, parent ion-water:+3: 499.64
