# Supplementary material for: Characterization of Prenylated C-terminal Peptides Using a Thiopropyl-based Capture Technique and LC-MS/MS
Source: Mol Cell Proteomics. 2020 Apr 13;19(6):1005–16. doi: 10.1074/mcp.RA120.001944 (PMC7261820; doi:10.1074/mcp.RA120.001944)
Supplement: Figure S4: msms spectrum of c-terminal peptide from Hras [file 158251_1_supp_495482_q7f3jn.pdf]

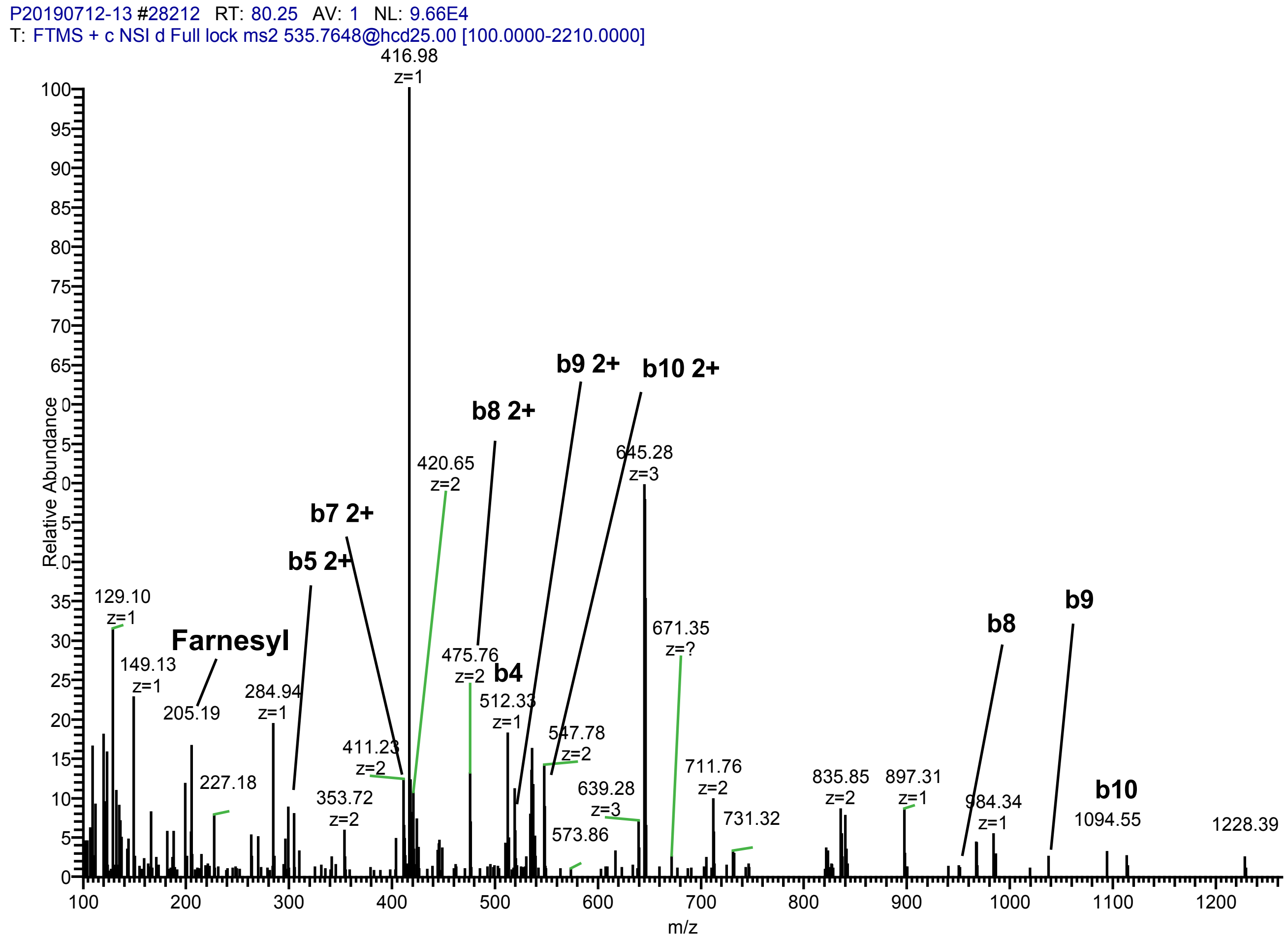

Figure S4. MS2 of **Hras** c-terminal peptide (L)RKLNPPDESGPGCMSCKC-Methyl+Farnesyl+4. M+4H+= 535.7648 from ms filter. Fragment masses are as follows (fragment:charge:mass): b5:+2: 305.19, b7:+2: 411.24, b8:+2: 475.76, b4:+1: 512.33, b9:+2: 519.27, b10:+2: 547.78, b8:+1: 950.51, b9:+1: 1037.54, b10:+1: 1094.56.
